# Transcriptome Profiling Analysis Reveals That Flavonoid and Ascorbate-Glutathione Cycle Are Important during Anther Development in Upland Cotton

**DOI:** 10.1371/journal.pone.0049244

**Published:** 2012-11-14

**Authors:** Jianhui Ma, Hengling Wei, Meizhen Song, Chaoyou Pang, Ji Liu, Long Wang, Jinfa Zhang, Shuli Fan, Shuxun Yu

**Affiliations:** 1 College of Agronomy, Northwest A&F University, Yangling, Shanxi, People's Republic of China; 2 State Key Laboratory of Cotton Biology, The Cotton Research Institute, Chinese Academy of Agricultural Sciences, Anyang, Henan, People's Republic of China; 3 Department of Plant and Environmental Sciences, New Mexico State University, Las Cruces, New Mexico, United States of America; Tsinghua University, China

## Abstract

**Background:**

Previous transcriptome profiling studies have investigated the molecular mechanisms of pollen and anther development, and identified many genes involved in these processes. However, only 51 anther ESTs of Upland cotton (*Gossypium hirsutum*) were found in NCBI and there have been no reports of transcriptome profiling analyzing anther development in Upland cotton, a major fiber crop in the word.

**Methodology/Principal Finding:**

Ninety-eight hundred and ninety-six high quality ESTs were sequenced from their 3′-ends and assembled into 6,643 unigenes from a normalized, full-length anther cDNA library of Upland cotton. Combined with previous sequenced anther-related ESTs, 12,244 unigenes were generated as the reference genes for digital gene expression (DGE) analysis. The DGE was conducted on anthers that were isolated at tetrad pollen (TTP), uninucleate pollen (UNP), binucleate pollen (BNP) and mature pollen (MTP) periods along with four other tissues, i.e., roots (RO), stems (ST), leaves (LV) and embryos (EB). Through transcriptome profiling analysis, we identified 1,165 genes that were enriched at certain anther development periods, and many of them were involved in starch and sucrose metabolism, pentose and glucuronate interconversion, flavonoid biosynthesis, and ascorbate and aldarate metabolism.

**Conclusions/Significance:**

We first generated a normalized, full-length cDNA library from anthers and performed transcriptome profiling analysis of anther development in Upland cotton. From these results, 10,178 anther expressed genes were identified, among which 1,165 genes were stage-enriched in anthers. And many of these stage-enriched genes were involved in some important processes regulating anther development.

## Introduction

The development of functional pollen and its releasing at appropriate stage to maximize pollination and fertilization are critical for plant reproduction and the creation of genetic diversity. These processes require cooperative interactions between gametophytic and sporophytic tissues within anther [1], [2]. Anther tissues consist of three layers: L1 layer that gives rise to epidermis and stomium; L2 layer that gives rise to archesporial cells, pollen mother cells (PMC), endothecium and middle wall layers; and L3 layer that gives rise to connective cells, vascular bundle and circular cell cluster. The L2 and L3 layers contribute to the formation of tapetum [3], which secretes nutrients and some secondary metabolites necessary for pollen development [4]. PMC undergo meiosis to form tetrads of haploid cells and each of them further develops into four microspores. The nucleus of each microspore divides into a vegetative and a generative cell nucleus, and the generative cell nucleus then divides into two sperm cells for double fertilization [5]. At the final stage, mature pollen grains are dispersed from anthers onto the stigma surface for fertilization.

Based on molecular studies, large numbers of genes related to pollen and anther development have been identified, especially in *Arabidopsis* and rice [1], [6]. For example, *DYSFUNCTIONAL TAPETUM1* (*DYT1*) encodes a putative basic helix-loop-helix (bHLH) transcription factor that is predicted to be downstream of *SPOROCYTELESS*/*NOZZLE* (*SPL*/*NZZ*) and *EXCESS MICROSPOROCYTES1*/*EXTRA SPOROGENOUS CELLS* (*EMS*1/*EXS*), and is required for the expression of *ABORTED MICROSPORES* (*AMS*) and *MALE STERILITY1* (*MS1*). It shows strong expression in tapetum and a low level of expression in meiocytes [7]. In the *dyt1* mutant of *Arabidopsis*, the tapetum becomes highly vacuolated, and the meiocytes lack a thick callose wall and eventually collapse [7]. In rice, the cytochrome P450 gene *CYP704B2*, which catalyzes the production of C16 and C18 *ω*-hydroxylated fatty acids, is predicted to be downstream of *Wall Deficient Anther1 (WDA1)* [8], [9]. The *cyp704B2* mutant exhibits swollen sporophytic tapetal layer, aborted pollen grains without detectable exine and undeveloped anther cuticles. Thus, *ω*-hydroxylated fatty acids appear to be essential for cuticle and exine formation during plant male reproductive and microspore development. However, only a few genes have been identified in the regulation of anther development in Upland cotton [10], [11].

Transcriptome profiling in *Arabidopsis*, maize and rice also has been performed to characterize the molecular mechanisms of pollen and anther development. For example, Honys and Twell used *Affymetrix* ATH1 genome arrays in *Arabidopsis* to identify 13,977 mRNA expressed in male gametophyte, 9.7% of which were specific to male gametophytes [12]. Ma et al. performed transcriptome profiling analyzing anther development in maize and identified many stage-specific and co-expressed genes [13]. Deveshwar et al. analyzed the anther transcriptome in rice and reported that approximately 22,000 genes were expressed in anthers. They also identified some genes contributing to meiosis and male gametophyte development from these data [14]. For more detailed information about anther, the differences in transcriptomes between tapetum and male gametophyte were found using laser microdissected cells in rice [4], [15]. Collectively, these studies have established a firm foundation for investigating the molecular mechanisms of pollen and anther development and sterility in plants.

However, there have been no transcriptome profiling analyzing anther development in Upland cotton, a major fiber crop in the world. Though the basic mechanisms of pollen and anther development could be cross-referenced, each species has its own peculiarity. Furthermore, most of these studies were carried out on self-pollinated and cross-pollinated plants. Pollen and anther development in Upland cotton, an often cross-pollinated crop, may somewhat differ from these other species. Thus, transcriptome profiling analysis of Upland cotton anthers is needed to provide a platform for investigating pollen and anther development and further analyzing male sterility. However, there are only 51 anther ESTs of Upland cotton deposited in the NCBI, and few molecular studies about anthers were carried out in this species.

Here, we described the construction of a normalized, full-length cDNA library from cotton anthers and transcriptome profiling analysis of anthers at different periods. In this study, we generated anther stage-enriched genes expression profiles in Upland cotton, and identified some important molecular processes regulating anther development and many genes that were involved in pollen mitosis and plant hormones regulation.

## Results

### Construction of the cDNA library

**Figure 1:**
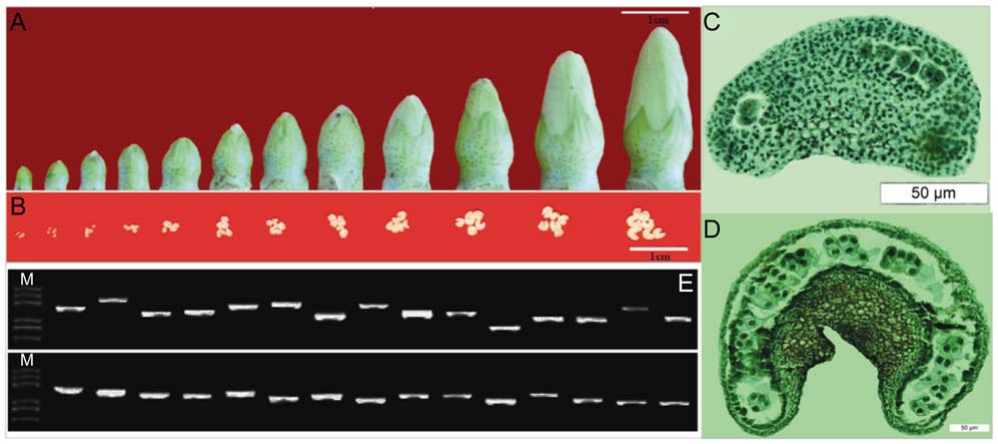
Construction of cDNA library from Upland cotton anthers. Flower buds (A) and the corresponding anther samples (B) were divided into 12 development stages. Anthers in stage 1 were at MEP (C) and anthers in stage 2 were at TTP (D). (E) Average insert size of the cDNA library was determined by PCR analysis of randomly selected clones. DL 2000 plus markers (lane M) were used for size determination.

Cell differentiation and dehiscence occur in a precise sequence that correlates with floral bud size during pollen and anther development [16], [17]. In this study, we divided CCRI 36 anthers into 13 development stages based on flower bud size and collected anther samples from each stage for cDNA library construction (Figure 1). Anthers samples in stage 1 and 2 were fixed in formalin-aceto-alcohol (FAA) for histological observation. Anthers in stage 1 were at meiosis period (MEP) and anthers in stage 2 were at tetrad pollen period (TTP) (Figure 1). The anthers of stage 13, mature pollen period (MTP), were collected at the day post-anthesis (0 dpa), when the flowers were still closed. Equal amounts of RNA from each anther stage were mixed to construct a normalized, full-length cDNA library.

The normalized, full-length cDNA library was estimated to contain about 1.2×10^6^ cfu/ml clones, similar to the normalized cDNA library constructed by Wang and Xia [18]. We randomly selected 60 clones for PCR amplification with M13 primers and estimated the fragment sizes by agarose gel electrophoresis. The size of all the fragments ranged between 1 and 3 kb, suggesting that the library contained relatively long cDNAs (Figure 1).

### EST sequencing and statistical analysis

**Figure 2:**
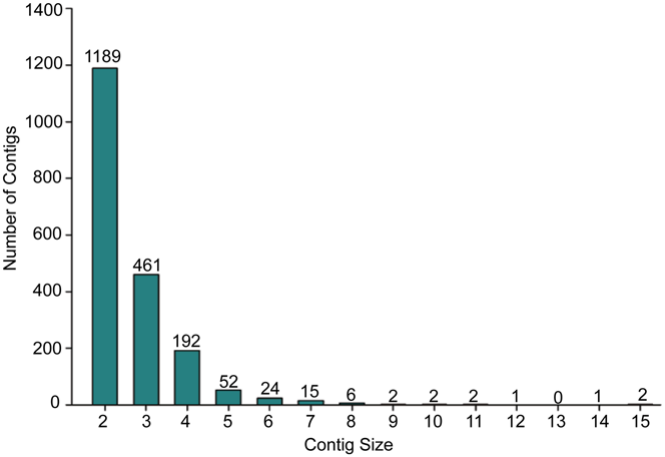
The distribution of ESTs in contigs. Assembly of 9896 ESTs resulted in 1949 contigs containing 5202 ESTs. The distribution of ESTs in each contig ranged between 2 and 15. Contig size represents the numbers of ESTs in each contig.

We sequenced 10,029 clones from this anther cDNA library from their 3′-ends. After removing the vector and low-quality sequences, we obtained 9,896 high-quality ESTs, which have been deposited in Genbank [DDBJ: 75889721–75899616]. These sequences were assembled into 6,643 unigenes including 4,694 singletons and 1,949 contigs (Table 1). The numbers of ESTs in contigs distributed between 2 and 15 (Figure 2). The average GC content of these unigenes was 40.33%, the lengths distributed between 111 bp and 1,901 bp, and most of them were longer than 600 bp.

**Table 1:** Summary of contig assembly.

Description	Number	Percentage
Total number of ESTs assembled	9896	
Number of contigs	1949	
Number of ESTs in contigs	5202	52.57
Number of ESTs as singletons	4694	47.43
Number of unique ESTs (unigenes)	6643	67.17

**Figure 3:**
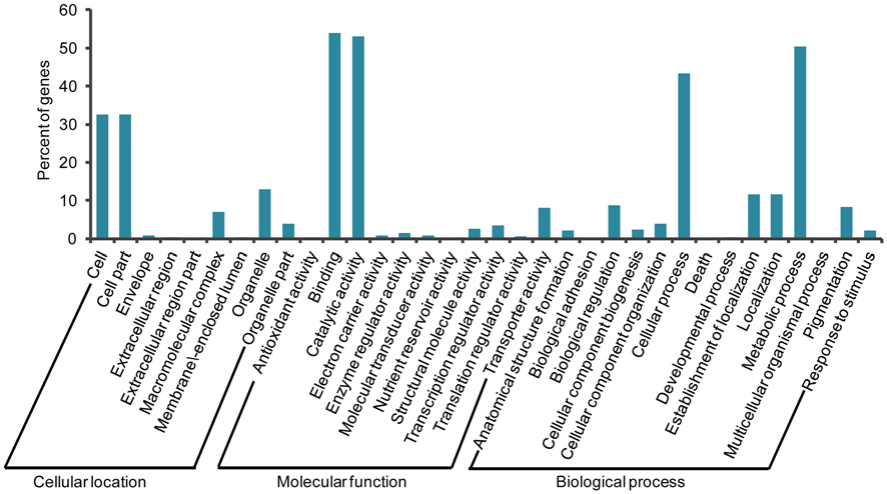
Gene ontology analysis of unigenes from the anther cDNA library. Unigenes were classified into three GO categories: cellular location, molecular function and biological process.

We compared these 9,896 high-quality ESTs with the ESTs and unigenes of Upland cotton available at Dana-Farber Cancer Institute (including 351,954 cotton ESTs, and 2,315 ESTs totally assembled into 117,992 unigenes). There were 3,754 ESTs (37.9%) with low homology (at least 25% of sequence with less than 95% of identity) to the existing ESTs and unigenes. This indicated that the ESTs from our cDNA library contained many novel sequences. These unigenes were classified into three gene ontology (GO) categories: cellular location, molecular function and biological process (Figure 3). For the cellular location category, large numbers of unigenes were categorized into cell and cell part. Under the molecular function category, the two most abundant sub-categories were binding and catalytic activity. For the biological process category, metabolic process and cellular process represented the major proportion.

Based on these 9,896 high-quality ESTs, together with 11,075 ESTs from a cDNA library [19] and 143 other anther ESTs [DDBJ: 75899617–75899759] which were from our previous studies on the same anther cDNA library, a total of 21,114 ESTs were assembled into 12,244 unigenes (Table S1). These unigenes were further used as reference gene set for DGE analysis in the following.

### Characterization of the sequenced DGE libraries

**Figure 4:**
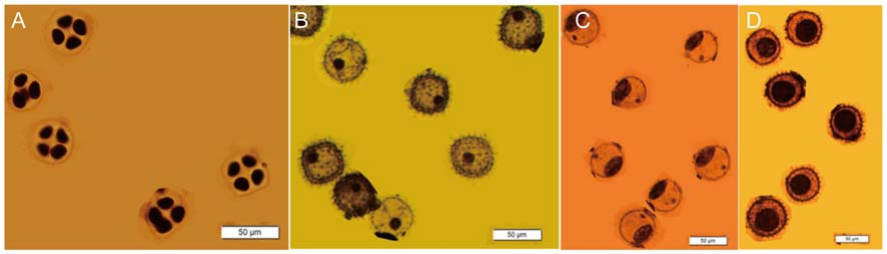
Development stages of the pollen grains in CCRI 040029. Pollen grains at TTP (A), UNP (B), early BNP (C) and mature BNP (D).

For transcriptome analysis of anther development in Upland cotton, we collected anthers at TTP, uninucleate pollen (UNP) and binucleate pollen (BNP) period from CCRI 040029, respectively (Figure 4). Anthers at mature pollen period (MTP) were collected at 0 dpa when the flowers were closed. Four other tissues including roots (RO), stems (ST), leaves (LV), and embryos (EB) were also harvested as a comparison. Thus, a total of 8 samples were prepared for Solexa sequencing and each generated 4.1–6.2 million raw reads. After removing the low quality reads, a total of 3.7–6.2 million reads were obtained and the number of reads with unique nucleotide sequences ranged from 116,087–758,811 in eight DGE libraries (Table S2). And the quality of these DEG libraries was acceptable (Table S2). To reveal the molecular events in eight DGE libraries, we mapped the tag sequences from each DGE library to the 12,244 unigenes, from which 36,760 reference tags were yielded and 97.15% of them were unambiguous (Table 2).

**Table 2:** CATG sites of reference genes.

	Number	Percentage
All genes	12244	
Genes with CATG site	11084	90.53
All reference tags	36760	
Unambiguous reference tags	35712	97.15

### Identification of anther stage-enriched genes

**Figure 5:**
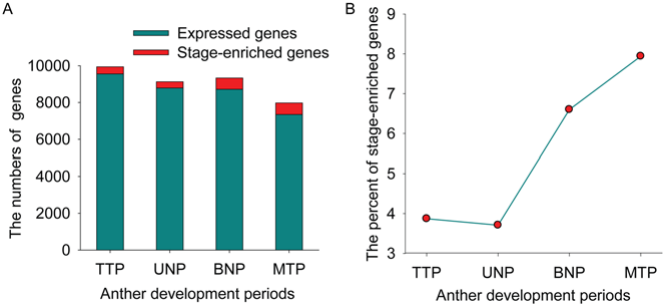
Distribution and proportion of anther stage-enriched genes in different stages of anther development. (A) Distribution of stage-enriched genes. (B) Proportion of stage-enriched genes.

From the DGE results, we found that there were 10,535 genes expressed in eight libraries, including 9,941 genes at TTP, 9,129 at UNP, 9,333 at BNP and 7,977 at MTP. The transcript diversity was significantly decreased from BNP to MTP (Figure 5). Anthers stage-enriched genes were identified using a combination of false discovery rate (FDR)≤0.001 and log2 ratio ≥2 by comparing the expression level in developing anthers (TTP, UNP, BNP and MTP) with other tissues (RO, ST, LV, EB). And 1,165 anther stage-enriched genes were found totally, including 245 genes with stage specific expression: 385 (3.87%) genes at TTP, 339 (3.71%) at UNP, 616 (6.60%) at BNP and 633 (7.94%) at MTP. The proportion of stage-enriched genes showed increase in later anther development stages (Table S3 and Figure 5).

**Figure 6:**
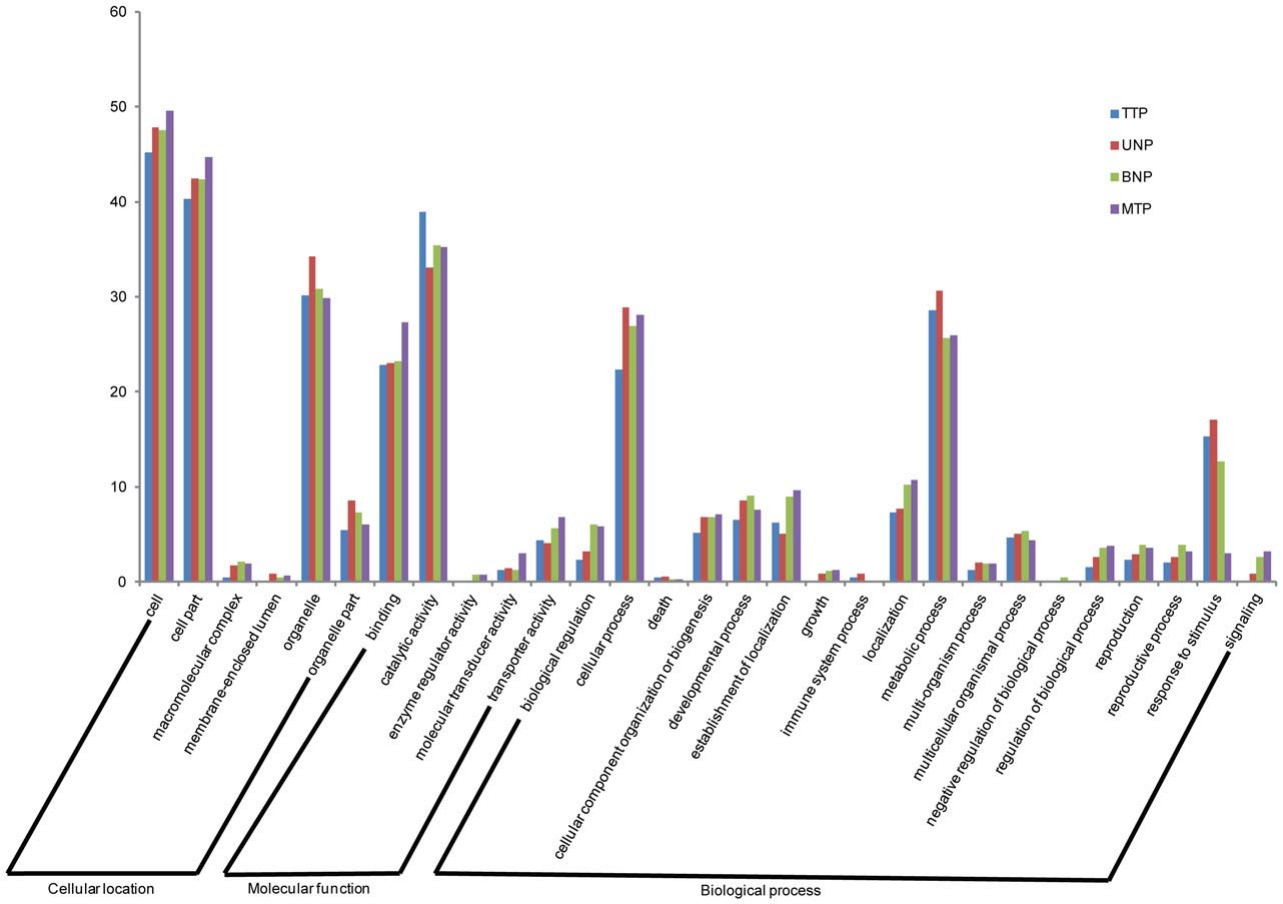
Gene ontology analysis of stage-enriched genes. Stage-enriched genes in each anther development stage were classified into three GO categories: cellular location, molecular function and biological process.

These stage-enriched genes were classified into three GO categories and they were concentrated toward the same GO sub-categories at four periods (Figure 6). And these main sub-categories were similar to the GO analysis of the complete anther cDNA library indicating that these processes should be important during anther development in Upland cotton. We further performed analysis of molecular function and cellular component term “enrichment status” and “hierarchy” on the stage-enriched genes at four periods. The results showed that there were some equally important processes at four anther periods, i.e., intrinsic to membrane and response to metal ion, and many important processes at certain period (Table S4). Previous research has identified many transcription factors (TF) that were related to pollen and anther development [7], [10], [20]. Here, we found 24 TF among these stage-enriched genes that were classified into 12 families and they were concentrated toward three families i.e., MADS, MYB and ERF (Table S5).

### Comparison of expression profiles associated with pollen and anther development between Upland cotton and *Arabidopsis*/rice

We predicted the protein sequences of these 1165 anther stage-enriched genes and performed BLASTx analysis between Upland cotton and *Arabidopsis*/rice (E-value≤1.0E^−5^, and identity ≥50%, Tair10 Proteins and version_7.0 Protein Sequences in Rice Genome Annotation Project). There were 812 homologous genes in *Arabidopsis* and 713 homologous genes in rice with these stage-enriched genes. Previous studies have focused on transcriptome profiling analysis of pollen and anther development and we identified 1691 pollen stage-enriched genes in *Arabidopsis* and 2201 anther stage-enriched genes in rice from these data (the expression level in anthers or pollen was more than 4 fold compared with other tissues) (Table S6) [12], [14]. Among these genes, only 155 in *Arabidopsis* and 120 in rice transcripts were overlapped with these anther stage-enriched genes in Upland cotton (Table S6). The lack of common stage-enriched genes could partly be attributed to the different processes during pollen and anther development between Upland cotton and *Arabidopsis*/rice.

### Validation of differentially expressed genes

**Figure 7:**
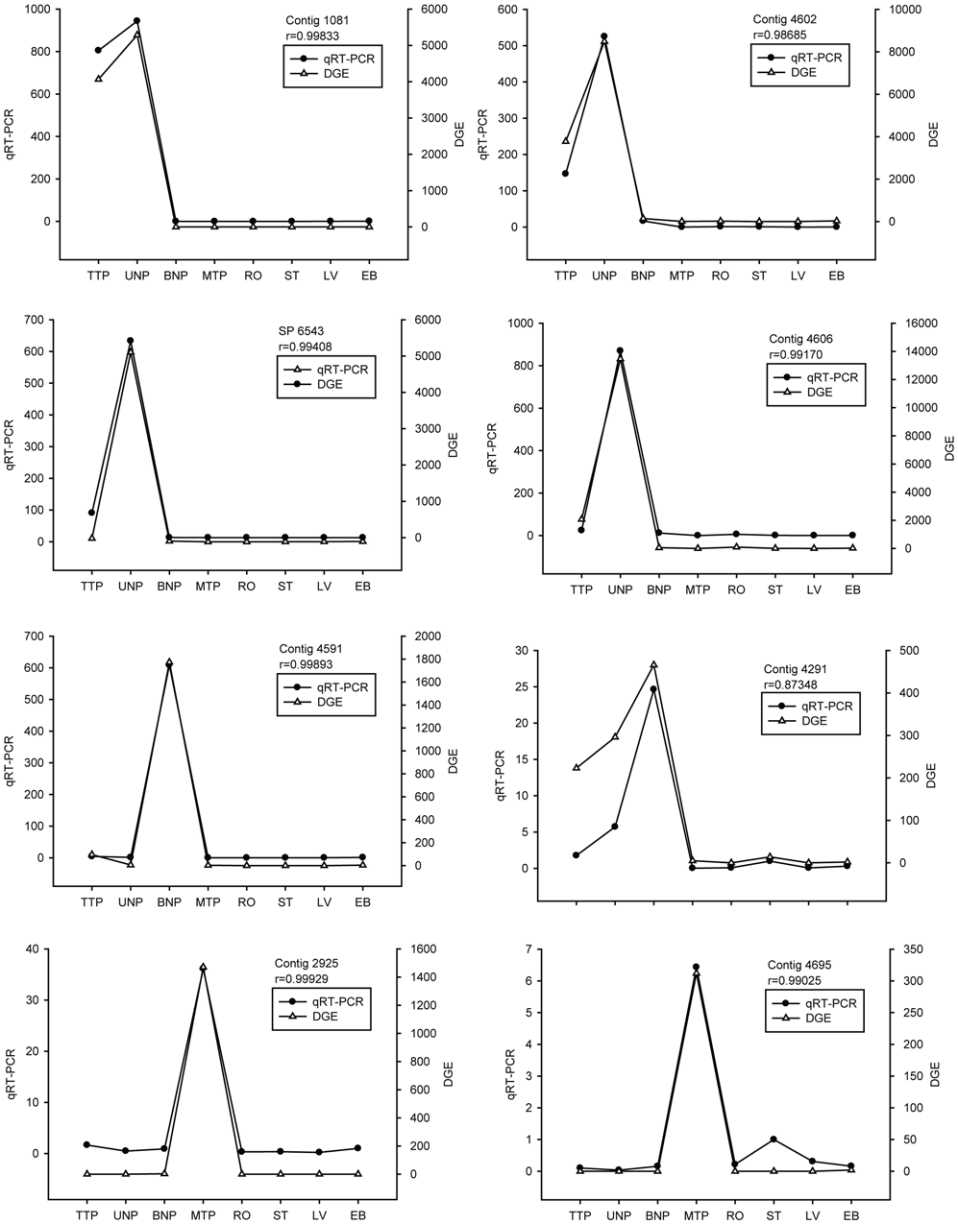
Confirmation of expression profiles of 26 transcripts by qRT-PCR. These charts showed the correlation between two types of expression profiles (DGE and qRT-PCR) for eight of the 26 transcripts. The correlation coefficients between the two expression profiles were more than 0.723 (P<0.05) for 23 transcripts, and more than 0.861 (P<0.01) for 17transcripts. (Detailed data is provided in Table S7).

To validate the candidate anther expressed genes, we performed quantitative real–time PCR (qRT-PCR) analysis. Twenty-six genes were randomly chosen from these anther stage-enriched genes, including three genes involved in flavonoid biosynthesis and four involved in ascorbate and aldarate metabolism. The expression levels of these examined genes ranged from 0 to 37,684. The profiles produced by qRT-PCR and the DGE results showed significant positive correlation for 23 of the 26 genes (p<0.05), indicating that 88% of the DGE expression data could be confirmed by qRT-PCR (Table S7 and Figure 7). Therefore, the gene expression revealed by DGEs should be reliably.

### Functional annotation of differentially expressed genes during anther development

We annotated the up-regulated genes (log2 ratio ≥2 and FDR≤0.001) to KEGG and identified significant pathways and the corresponding genes using a threshold of Q-value≤0.05 (Table S8). Comparing with other tissues (RO, ST, LV and EB), starch and sucrose metabolism, pentose and glucuronate interconversion were significant at TTP, BNP and MTP. Two pathways related to antioxidant production, flavonoid biosynthesis and ascorbate and aldarate metabolism, were significant at different anther development periods. The pathway of flavonoid biosynthesis was significant at TTP and UNP (early development periods), and the pathway of ascorbate and aldarate metabolism was significant at BNP and MTP (later development periods). During anther development, two mitosis related pathways, DNA replication and base excision repair, were significant at UNP compared with MTP. We also studied the expression pattern of phytohormone response genes through tags mapping.

#### Flavonoid biosynthesis

**Figure 8:**
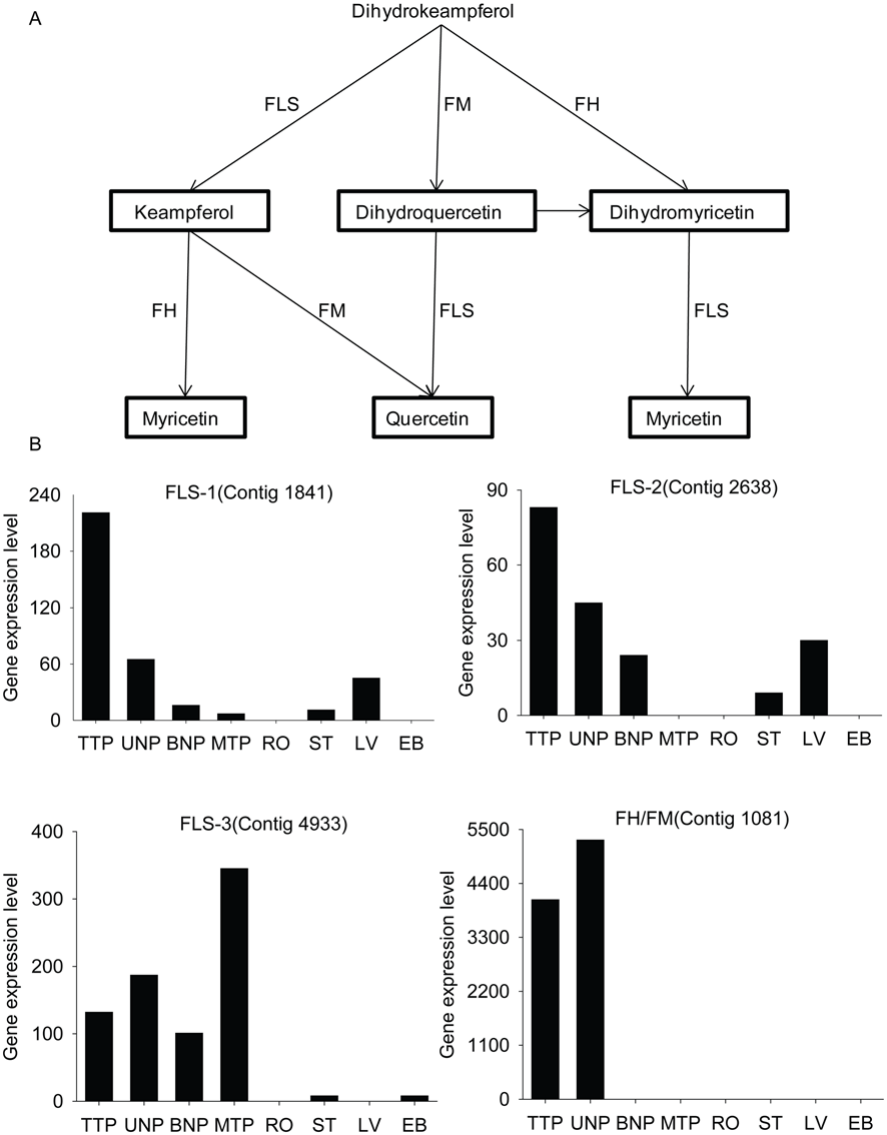
The up-regulated process in flavonoid biosynthesis and the expression levels of the corresponding genes. (A) The up-regulated process in flavonoid biosynthesis. Myricetin and quercetin were the final produces in this process. (B) Expression levels of the corresponding genes in DGE libraries. Abbreviations: FLS, flavonol synthase; FM, flavonoid 3′-monooxygenase; FH, flavonoid 3′,5′-hydroxylase.

Flavonoids are free radical scavengers and also are components of pollen coat [21], [22]. During anther development in Upland cotton, the pathway of flavonoid biosynthesis was significant at TTP and UNP relative to other tissues (RO, ST, LV and EB). Many anther stage-enriched genes related to the biosynthesis of quercetin, kaempferol, myricetin, dihydromyricetin and dihydroquercetin were found at TTP and UNP (Table 3). Quercetin and myricetin were the final products of this process, and they should be the main flavonoids in cotton anthers during the early anther development stages (Figure 8). In addition, these anther stage-enriched genes had strong homologs in *Arabidopsis*, and the homologous genes of contig723, contig1081 and SP16499 were associated with pollen coat formation (Table 3) [22]–[24]. Hsieh and Huang [22] have reported that flavonoids would be deposited onto the pollen coat after tapetum lysis in *Brassica*. Here, flavonoids also appeared to be important components of the pollen coat in Upland cotton (Table S9).

**Table 3:** Stage-enriched genes in flavonoid biosynthesis and in ascorbate and aldarate metabolism.

Unigenes	EC NO.	Annotation	Periods	Homology	References
**Flavonoid biosynthesis**					
Contig1841	1.14.11.23	Flavonol synthase	TTP	AT5G08640	
Contig2638	1.14.11.23	Fe(II)-dependent oxygenase	TTP	AT4G10490	
Contig4933	1.14.11.23	Flavonol synthase	TTP/UNP	AT5G08640	
Contig723	2.3.1.74	Chalcone synthase	TTP/UNP	AT5G13930	[22]
Contig4115	1.14.13-	Cytochrome P450	TTP/UNP	AT5G09970	[28]
Contig128	1.1.1.234	bifunctional dihydroflavonol 4-reductase flavanone 4-reductase	UNP	AT2G45400	
Contig1081	1.14.13.21	Cytochrome P450	TTP/UNP	AT1G01280	[23]
	1.14.13.88				
SP16499	2.3.1.74	Chalcone and stilbene synthases	TTP/UNP	AT1G02050	[24]
**Ascorbate and aldarate metabolism**					
SP1354	1.2.1.3	Aldehyde dehydrogenase	MTP	AT4G36250	
Contig218	1.2.1.3	Aldehyde dehydrogenase	BNT/MTP	AT1G54100	
Contig3580	1.11.1.11	Plant ascorbate peroxidase	BNT/MTP	AT1G07890	
Contig3634	1.11.1.11	Plant ascorbate peroxidase	BNT/MTP	AT1G07890	
Contig4658	1.10.3.3	Oxidoreductase activity	BNT/MTP	AT3G13400	[29]
Contig5009	1.10.3.3	Oxidoreductase activity	BNT/MTP	AT1G55570	[29]
Contig5036	1.10.3.3	Oxidoreductase activity	BNT/MTP	AT3G13390	[29]
SP15395	1.10.3.3	Oxidoreductase activity	BNT	AT4G12420	
Contig3413	1.10.3.3	Oxidoreductase activity	BNT/MTP	AT1G55570	[29]
Contig2930	1.13.99.1	Myo-inositol oxygenase gene family	BNT	AT2G19800	
Contig4099	1.13.99.1	Myo-Inositol oxygenase gene family	BNT/MTP	AT5G56640	
Contig1156	1.1.1.22	UDP-glucose GDP-mannose dehydrogenase	BNT	AT5G15490	
SP13121	5.1.3.18	NAD-dependent epimerase/dehydratase	BNT/MTP	AT2G47650	

#### Ascorbate and aldarate metabolism

**Figure 9:**
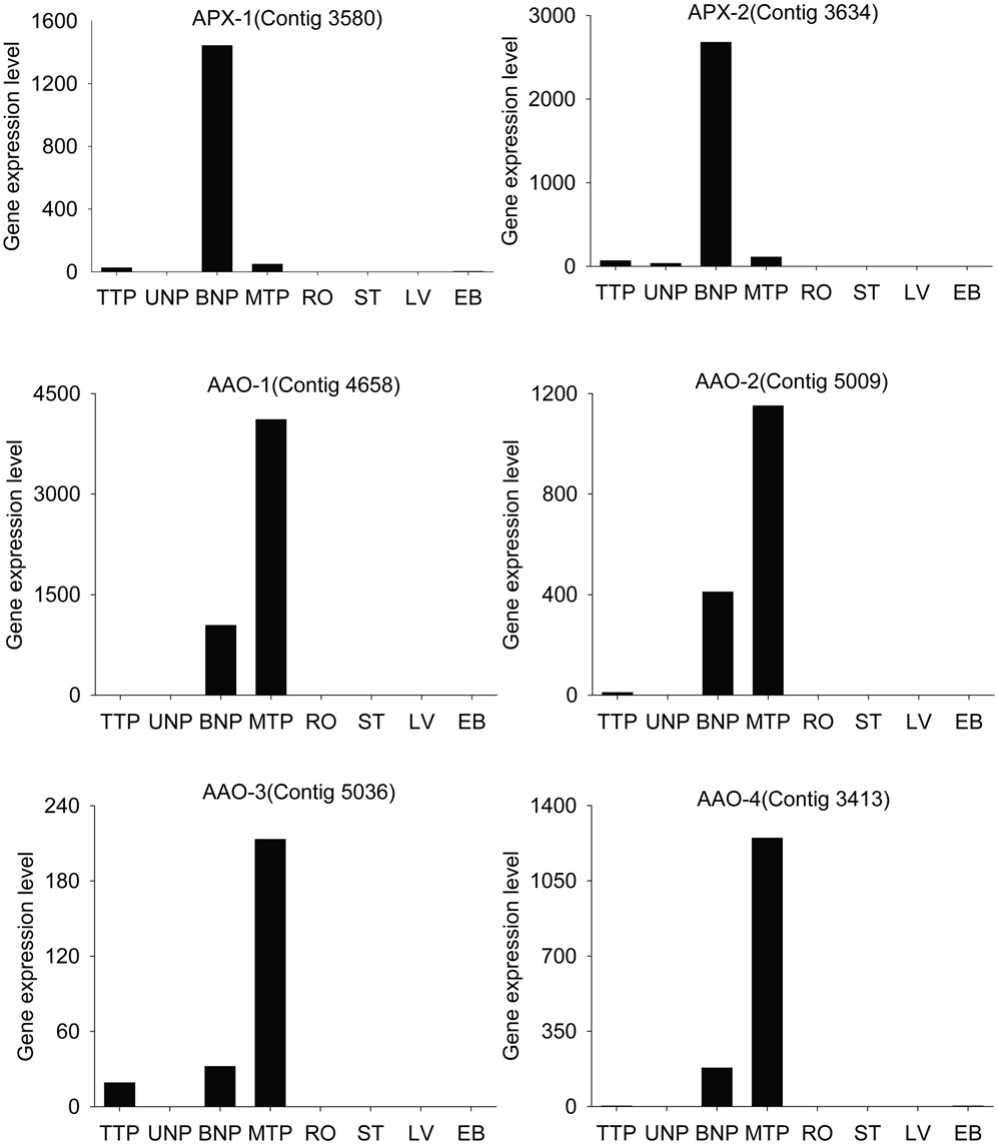
The expression levels of stage-enriched genes involved in ascorbate-glutathione cycle.

**Figure 10:**
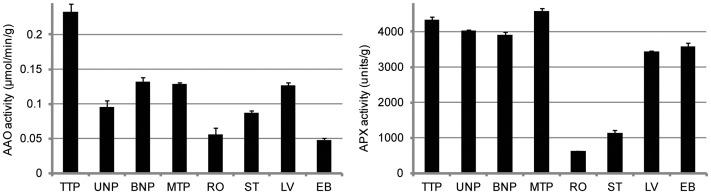
Enzymatic activity of AAO and APX.

Ascorbic acid (AsA) is an essential member of antioxidants and also has functions in other enzymatic reactions and cellular processes, such as growth and mitosis [25], [26]. The pathway of ascorbate and aldarate metabolism was significant at BNP and MTP relative to RO, ST, LV and EB. And many anther stage-enriched genes at BNP and MTP were related to L-ascorbate oxidase [AAO; EC 1.10.3.3] and L-peroxidase [APX; EC 1.11.1.11], which were in the ascorbate-glutathione cycle, one of the important processes for free radical detoxification (Figure 9 and Table 3) [27]. We also assayed AAO and APX activities in the eight samples and they had higher activities during anther development than in other tissues (Figure 10), confirming that the ascorbate-glutathione cycle is important in anthers of Upland cotton (Table S9).

#### Mitosis

During anther development in Upland cotton, all pollen grains were at UNP in stage 5 and developed to BNP in stage 6 (Figure 4). Thus, rapid mitosis should be occurred at UNP to form binucleate pollen grains. Our results indicated that the two mitosis related pathways, DNA replication and base excision repair, were significant at UNP relative to MTP. Some of the genes involved in the two pathways were also up-regulated at BNP compared with MTP. The pollen grains were all binucleate in stage 7 and developed to trinucleate in stage 14, when their pollen tubes germinated [30]. Thus, mitosis process should have been commenced in the early stage of BNP (Table S9).

#### Phytohormones

Phytohormones play an important role in regulating anther development [1], [31]. In this study, the expression pattern of 104 phytohormone response genes were obtained in the eight DGE libraries by tag mapping (Table S10). A total of 23 of these genes were related to *DELLA* which was as the gibberellins negative regulators [31]. And most of them were up-regulated at TTP and UNP, indicating that GA should play its role mainly in the later development stages. We also identified 81 auxin and jasmonic acid response genes, most of which were also up-regulated at TTP and UNP. Thus, these two hormones appeared to be important in the early stages of anther development.

## Discussion

Previous plant biology studies have used transcriptome analysis to investigate the molecular mechanisms of pollen and anther development. However, there have been no reports of transcriptome profiling analyzing anther development in Upland cotton. In this study, 9,896 high quality ESTs were sequenced and assembled into 6,643 unigenes from a full-length, normalized cDNA library of Upland cotton anthers. They, together with other anther-related ESTs sequenced from the 3′-end in our group, were further assembled to 12,244 unigenes, which were used as an ideal reference gene set for transcriptome profiling analysis of Upland cotton anthers isolated at TTP, UNP, BNP, MTP along with other tissues (RO, ST, LV and EB) using DGE. And this method has been used for many plants, such as maize [32], cucumber [33], cotton [34] and grape [35]. From these data, we found 10,535 genes that were expressed in eight libraries, 10,178 of which were expressed in anthers. We also identified 1,165 anther stage-enriched genes and some important biological processes during anther development in Upland cotton. To our knowledge, Curtiss et al. have recently used anthers and ovules of Pima cotton (*G. barbadense*) at 0 dpa to compare differentially expressed genes between Pima S-1 and isogenic 57-4 [36]. And this is the first study to construct an anther cDNA library of Upland cotton and investigate transcriptional changes during anther development in this species. The data presented here will be a useful platform for studies of the molecular aspects of anthers in Upland cotton.

Flavonoids are plant secondary metabolites that have a vital role in the fertility of higher plants. Mutants of maize and petunia with blocked chalcone synthase, a key enzyme in flavonoid biosynthesis, are sterile [37]–[39]. Flavonoids are also present in the pollen coat to protect pollen grains from ultraviolet irradiation damage and serve as free radical scavengers during anther development [21], [22]. In our study, the pathway of flavonoid biosynthesis was significant at TTP and UNP relative to RO, ST, LV and EB. Homologs and the expression patterns of these stage-enriched flavonoid biosynthesis genes were found in *Arabidopsis* and rice [12], [14]. However, only two homologs were expressed in *Arabidopsis* pollen, which differed from the anther transcriptomes of Upland cotton and rice. Taking the data of pollen and anther transcriptomes together, we conjectured that flavonol biosynthesis and the corresponding genes should have high activities in anther wall, as described by Hsieh and Huang [22]. In addition, the flavonoids would be deposited on the pollen coat upon tapetum lysis, between TTP and BNP [22], [40], so the corresponding flavonoids genes should be highly expressed in early anther development stages, as indicated by our data. We expected that some similar processes should be occurred during pollen and anther development among plants.

AsA is an important radical scavenger and also has roles in some cellular processes during anther development. The *vtc1* mutant of *Arabidopsis* that cannot produce AsA from mannose is sterile under 16-h day: 8-h night conditions [21], [41], [42]. Our results indicated that many anther stage-enriched genes were involved in the ascorbate-glutathione cycle at BNP and MTP and the AAO and APX had high activities in anthers. During anther development, many potential environmental stressors could lead to the accumulation of reactive oxygen species (ROS). The ascorbate-glutathione cycle appeared to play an important role to detoxify ROS and maintain anther development in Upland cotton. Homologous genes were also found in *Arabidopsis*/rice, but only one was enriched in anthers of rice and four were enriched in pollen of *Arabidopsis*. This indicated that some significant molecular processes should be somewhat different in the processes of pollen and anther development among plants.

In *Arabidopsis* and rice, the nucleus of pollen grains could be seen clearly from uninucleate to trinucleate pollen periods by 4′, 6-diaminophenylindole (DAPI) staining [12], [43]. However, the pollen grains of Upland cotton have a thickened pollen coat and denser cytoplasm. And the pre-treatment by acids (15% chromic acid : 10% nitric acid : 5% hydrochloric acid) was required to observe pollen development [44]. Even then, the pollen nucleuses were not visible owing to the dense cytoplasm from the mature BNP to MTP (Figure 4). In addition, the pollen grains of Upland cotton developed to trinucleate period on the day of blooming, when the pollen tubes germinated, unlike the processes in *Arabidopsis* and rice [30]. These different processes may be related to the differences of transcriptome profiling between them and Upland cotton.

In this study, we have identified many unique ESTs from an anther cDNA library and provided detailed descriptions of gene expression patterns at different periods of anther development. We also revealed some interesting molecular features during anther development in Upland cotton. In summary, our trancriptome analysis of anthers laid a good foundation for investigation of pollen/anther development and further study of the sterility mechanisms in Upland cotton.

## Materials and Methods

### Plant material

Lai et al. have constructed a cDNA library using the Upland cotton cultivar CCRI 36 [19]. To study the expressed genes in anthers and combine the ESTs from two cDNA libraries better, we used the anthers of CCRI 36 to construct anther cDNA library. For cDNA library construction, we defined 13 anther development stages from CCRI 36 based on flower bud size. Anther samples were collected from each stage for RNA extraction. And the Anthers in stage 1 and 2 were fixed in FAA for histological observation.

Furthermore, to analyze anther development in upland cotton and make a firm foundation for further analysis of male sterility in Upland cotton, we chose CCRI 040029, the wild-type of a photo-periodically sensitive genetic male sterile mutant, to analyze the transcriptome changes during anther development. Anther samples at TTP, UNP, BNP and MTP were collected for DGE sequencing. For a comparison, the RO, ST, LV, and EB from CCRI 040029 were also collected in early inflorescence.

### Histological observations

For longitudinal section observation, anther samples from CCRI 36 were fixed in FAA and dehydrated in an ethanol series. The samples were then embedded in resin. Longitudinal sections were cut using an ultramicrotome (Leica RM2265, Germany), stained by safranin with a fast green counterstain and photographed using light microscopy (Olympus DP72, Japan). To observe anther development in CCRI 040029, pollen grains from each stage were squeezed out and dissolved in mixed acids (15% chromic acid, 10% nitric acid, 5% hydrochloric acid) and 1% aceto carmine [44]. The observation process was conducted using light microscopy (Olympus DP72, Japan).

### RNA extraction

Samples for RNA extraction were immediately immersed in liquid nitrogen and stored at −80°C until RNA extraction. We extracted RNA using a modified CTAB method [45]. RNA samples with A_260_/A_280_ ratios between 1.8 and 2.0 and A_260_/A_230_ ratios more than 1.5 were considered acceptable.

### cDNA library construction and EST analysis

Equal amounts RNA in each anther stage from CCRI 36 were mixed, and this mixture was used to construct the cDNA library. The processes of cDNA library construction and EST analysis followed Lai et al. [19].

We compared the ESTs with database version 11.0 of the Dana-Farber Cancer Institute Cotton Gene Index (http://compbio.dfci.harvard.edu/cgi-bin/tgi/gimain.pl?gudb=cotton). An EST was considered as new if it had at least 25% of sequence with less than 95% of identity with any other EST or unigene in the public EST database [46]. And the molecular function and cellular component term “enrichment status” and “hierarchy” of anther stage-enriched genes were analyzed using agriGO (http://bioinfo.cau.edu.cn/agriGO/) [47].

### DGE sequencing and tag annotation

At least 6 µg of total RNA (>300 ng·µL^−1^) from each sample of CCRI 040029 was sent to the “Beijing Genomics Institute” (BGI, Shenzhen, China) for high-throughput Solexa sequencing. First, poly(A)-containing mRNA molecules were purified from total RNA using poly(T) oligo-attached magnetic beads. First- and second-strand cDNA was synthesized. While on the beads, double-stranded cDNA was digested with NlaIII endonuclease to produce a bead-bound cDNA fragment containing the sequence from the 3′-most CATG to the poly(A)-tail. cDNA fragments with 3′-ends were purified by magnetic bead precipitation, and Illumina adapter 1 was added to the 5′-ends. The junction of Illumina adapter 1 and the CATG site is the recognition site of Mmel, which cleaves 17 bp downstream of the CATG site and produces 21 bp tags. Illumina adapter 2 was ligated to the 3′-end of the cDNA tag after removing the 3′ fragments by magnetic bead precipitation. These adapter-ligated cDNA tags were enriched using PCR primers that anneal to the adaptor ends. The resulting 85-bp fragments were purified by 6% TBE PAGE. Fragments were then digested and the single-chain molecules were fixed onto the Solexa Sequencing Chip. Four-color fluorescent labeled nucleotides were added to the chip, and fragments were sequenced by synthesis using an Illumina Genome Analyzer.

Useless tags (3′-adaptor sequences, empty reads, low-quality sequences, tags that were too long or too short and tags with only one copy number) were deleted and each clean-tag library consisted of 21-bp fragments. These clean tags were matched to the reference gene set. The number of annotated clean tags for each gene was normalized to number of transcripts per million clean tags, a standard method used in DGE analysis [48].

### Statistical evaluation of DGE libraries

An algorithm developed by Audic and Claverie was used to identify differentially expressed genes between libraries [49]. The threshold of P value is determined by controlling the FDR in multiple tests. The genes, which differentally expressed between libraries, were identified using the combination of FDR≤0.001 and log2 ratio ≥2. The differentially expressed genes between DGE libraries were mapped to the KEGG database. And the significant pathways were identified under a threshold of Q-value≤0.05.

### qRT-PCR

Reverse transcription reactions were performed using 4.0 µg RNA with SuperScriptIII reverse transcriptase (Invitrogen, USA). Primers were designed using Oligo6 and were synthesized by SANGON (Shanghai, China). Reactions were carried out using SYBR Green PCR Master Mix (Roche Applied Science, Germany) on an ABI 7500 real-time PCR system (Applied Biosystems, USA) with three replicates, and the amplification of 18S rRNA was used as an internal control for data to normalization. Reaction volumes were 25 µL containing 12.5 µL SYBR Green PCR Master Mix, 9.5 µL distilled/deionized H_2_O, 1 µL primers and 2 µL cDNA. Amplification reactions were initiated with a pre-denaturing step (95°C for 10 min), followed by denaturing (95°C for 10 s), annealing (60°C for 35 s) and extension (72°C for 35 s) for 40 cycles. Data were processed using the 2^−ΔΔCt^ method [50].

### Determination of AAO and APX activity

AAO and APX activities were assayed using a modified spectrophotometric method [51], [52]. For AAO extraction, sample (0.5 g) was mixed and homogenized with 5 mL phosphate buffer (0.1 M, pH 6.0, containing 0.5 mM EDTA and 1 M NaCl). The homogenates were centrifuged (11,000× *g* for 30 min) at 4°C. Each reaction contained 2500 µL sodium phosphate buffer (0.1 M, pH 6.0, containing 0.5 mM EDTA), 400 µL extract, and 100 µL 1.5 mM ASA substrate solution. One unit of AAO activity was defined as the number of µmol of ASA catalyzed per min (25°C, pH 6.0) based on absorption at the wavelength of 265 nm.

For APX extraction, sample (0.5 g) was mixed and homogenized with 5 mL phosphate buffer (0.05 M, pH 7.8, containing 2% PVP, 0.1 mM EDTA, 0.1 mM ASA, 0.1 mM dithiothreitol, 0.1 mM reduced glutathione, and 0.5 mM MgCl_2_). The homogenates were centrifuged (11,000× *g* for 20 min) at 4°C. Each reaction contained 2700 µL sodium phosphate buffer (0.05 M, pH 7.0, containing 0.1 mM EDTA), 100 µL extract, 100 µL 7.5 mM ASA, and 100 µL 300 mM H_2_O_2_. APX activity was determined by the reduction of H_2_O_2_ based on absorption at the wavelength of 290 nm.

## Supporting Information

Table S1 The ESTs name collected from another cDNA library and reference unigenes assembled from the two cDNA libraries.
(XLS)

Table S2 The summary information and quality evaluation of eight DGE libraries.
(XLS)

Table S3 Genes expression pattern in DGE libraries and the stage-enriched genes.
(XLS)

Table S4 The analysis of the molecular function and cellular component term enrichment status and hierarchy of anther stage-enriched genes.
(XLS)

Table S5 Transcription factor analysis.
(XLS)

Table S6 Homology analysis compared with rice and Arabidopsis.
(XLS)

Table S7 The correlation between DGE and qRT-PCR and the primers for qRT-PCR.
(XLS)

Table S8 The significant pathways through compared with each other among these eight DGE libraries.
(XLS)

Table S9 Stage-enriched genes in significant pathways.
(XLS)

Table S10 The sequence and expression pattern of phytohormones related genes.
(XLS)
